# Phobic anxiety disorders among patients with acne vulgaris at a national dermatology hospital in Vietnam: Prevalence, clinical phenotype, and associated factors

**DOI:** 10.1371/journal.pone.0357803

**Published:** 2026-09-10

**Authors:** Nguyen Van Tuan, Vu Thai Ha, Mai Son Duong

**Affiliations:** 1 Hanoi Medical University, Vietnam; 2 National Institute of Mental Health - Bach Mai Hospital, Vietnam; 3 National Hospital of Dermatology and Venereology, Vietnam; 4 Bien Hoa National Institute of Forensic Psychiatry, Dong Nai, Vietnam; Bogomolets National Medical University: Nacional’nij medicnij universitet imeni O O Bohomol’ca, UKRAINE

## Abstract

**Background:**

Acne vulgaris is associated with psychological morbidity, but clinically diagnosed phobic anxiety disorders (PADs) have been little studied in dermatology settings.

**Methods:**

The analysis comprised 280 patients with acne vulgaris recruited by convenience sampling at a national dermatology hospital in Hanoi, Vietnam, from December 2025 to June 2026. A psychiatrist established PAD diagnoses using ICD-10 clinical criteria; Fear Questionnaire scores were used descriptively to characterize phobic symptoms among diagnosed cases. Multivariable logistic regression estimated adjusted odds ratios (aORs) and 95% confidence intervals (CIs) for factors associated with PADs.

**Results:**

PADs were diagnosed in 69 of 280 participants (24.6%; 95% CI, 20.0%–30.0%). Social phobia affected 64 participants (22.9% of the sample; 92.8% of PAD cases), specific phobia affected 26 (9.3%), and agoraphobia affected one (0.4%); subtypes could co-occur. In the exploratory adjusted model, PADs were associated with study-specific anxiety-proneness rating (aOR, 6.90; 95% CI, 2.92–16.32), physical comorbidity (aOR, 4.70; 95% CI, 2.30–9.60), acne scarring (aOR, 2.29; 95% CI, 1.04–5.05), and poor treatment response (aOR, 5.06; 95% CI, 2.20–11.62). Clinician-rated severe acne was not associated with PADs after adjustment.

**Conclusions:**

In this tertiary-care sample, PADs—predominantly social phobia—were common. Associations with scarring, treatment response, physical comorbidity, and study-specific anxiety-proneness warrant confirmation in prospective multicentre studies using standardized psychosocial and treatment-response measures.

## 1. Introduction

Acne vulgaris is a common inflammatory skin disease with effects that extend beyond cutaneous symptoms. It occurs most often during adolescence and early adulthood, when appearance, peer acceptance, self-image, and social functioning may be especially salient. Acne affects approximately 9% of the global population, with the greatest burden in younger age groups [1,2]. Its chronic course, facial visibility, risk of scarring, and repeated treatment can impose physical and psychosocial burdens [1,3]. Acne is therefore increasingly considered within a psychodermatological framework linking skin disease, self-perception, and social functioning.

The psychological burden of acne is clinically important. Dermatology studies have documented anxiety, depressive symptoms, impaired quality of life, and social discomfort among patients with acne [4]. A meta-analysis of 42 studies found associations of acne with both depression and anxiety, with heterogeneity by age, clinical setting, and geographic region [5]. Population-based evidence has also linked acne with an increased subsequent risk of major depression [6]. Together, these findings support attention to mental health in dermatology care, particularly for patients with persistent, visible, or treatment-resistant disease.

Most previous research, however, has examined general anxiety or depressive symptoms rather than clinician-diagnosed phobic anxiety disorders. PADs are characterized by disproportionate fear, anticipatory anxiety, and avoidance of specific objects, situations, or social contexts, and include social phobia, agoraphobia, and specific phobias [7,8]. In acne, fear of scrutiny or rejection may contribute to avoidance of social interaction, public appearance, school, or work. Experimental evidence indicates that acne can elicit stigmatizing judgments in social and professional scenarios [9], and one case-control study reported a high frequency of social phobia among patients with acne [10]. These observations provide a rationale for examining disorder-level phobic phenotypes rather than nonspecific distress alone.

Scarring, persistent disease, unsatisfactory treatment response, and acne-related life impact may be relevant to phobic anxiety. Visible sequelae can persist after active lesions improve, while repeated or unsuccessful treatment may prolong appearance-related distress and uncertainty [1,9,11–13]. Psychological vulnerability and somatic or psychiatric comorbidity may further influence how acne is experienced [5,11,12]. A biopsychosocial framework can therefore examine whether PADs are associated with clinician-rated severity alone or also with scarring, treatment experience, comorbidity, and study-specific psychosocial characteristics.

Clinician-diagnosed PAD phenotypes among Vietnamese acne outpatients have been poorly described, and evidence from Southeast Asian dermatology settings remains limited. Psychodermatology reviews emphasize that psychological burden may be overlooked in routine acne care and that dermatology clinicians may be an important first point of recognition and referral [11,12]. Studying PADs in a Vietnamese tertiary-care setting may therefore add clinically relevant evidence while requiring caution about referral-setting generalizability.

We therefore aimed to (1) estimate the proportion of patients with PADs in a hospital-based acne outpatient sample; (2) describe PAD subtypes and Fear Questionnaire profiles; and (3) examine dermatological, treatment-related, somatic, and psychosocial factors associated with PADs. We hypothesized that PADs would be associated not only with clinician-rated acne severity but also with acne scarring, treatment response, physical comorbidity, and psychological vulnerability.

## 2. Methods

### 2.1. Study design and setting

We conducted a cross-sectional study at the National Hospital of Dermatology and Venereology in Hanoi, Vietnam. Participants were recruited from December 2025 through June 2026 under the approved protocol. We report the study in accordance with the Strengthening the Reporting of Observational Studies in Epidemiology (STROBE) guidance for cross-sectional studies [14].

### 2.2. Participants

Eligible participants were patients receiving care at the study hospital during the recruitment period who had dermatologist-diagnosed acne vulgaris according to Vietnamese Ministry of Health guidance, could complete a face-to-face interview and understand the study questionnaire, and agreed to participate.

Patients were excluded if they had a previously diagnosed psychiatric condition whose current severity substantially interfered with understanding or completing the assessment (operationally termed a severe psychiatric disorder in the protocol); an acute systemic illness, severe infection, or acute dermatological complication that could affect psychological status or concentration; or if they withdrew before completing the study procedures. This eligibility criterion was distinct from concurrent anxiety/depressive symptoms recorded among participants who remained able to complete the study assessment.

A convenience sampling strategy was used. The minimum sample size was calculated with the single-population proportion formula:


$$\begin{array}{c} n=Z^{\text{2}}\times \frac{p(\text{1}-p)}{d^{\text{2}}} \end{array}$$

For a two-sided α of 0.05, Z was 1.96, the absolute precision d was 0.06, and the anticipated proportion p was 0.457, based on the social-phobia frequency reported by Bez *et al.* [10]. The resulting minimum sample size was 265. The final analysis included 280 participants with an available PAD assessment.

The available recruitment archive did not retain a complete screening log. Consequently, the numbers of patients approached, declining participation, or excluded before enrollment and a response rate could not be reconstructed reliably; unavailable counts were not inferred.

### 2.3. Data collection

Data were collected through a structured study case-report form, face-to-face interviews, dermatologist-led clinical examination, psychiatrist-led assessment, and medical-record review. The case-report form captured demographic and socioeconomic characteristics, medical and psychiatric history, acne characteristics and treatment, acne-related life impact, phobic symptoms, and avoidance behaviours. Several psychosocial characteristics were study-specific categorical case-report-form items rather than validated psychometric scales; operational definitions are summarized in S4 Table.

Dermatological variables were recorded during dermatologist-led assessment using clinical examination, participant interview, and available medical records. Variables included age at acne onset, acne duration, lesion type and distribution, severity, scarring and other sequelae, previous and current treatment, systemic corticosteroid and isotretinoin use, follow-up visits, treatment changes during the preceding 12 months, and treatment response. Acne severity was recorded as mild, moderate, or severe using a three-level clinician global assessment informed by Investigator’s Global Assessment (IGA) principles [13,15]; the analytic archive did not contain a formal numerical IGA score. At the study visit, treatment response was recorded on the dermatology case-report form as marked, partial, or none. This was a pragmatic study-specific clinical classification rather than a standardized longitudinal outcome; no prespecified lesion-count reduction, numeric IGA change, or validated patient-satisfaction threshold was used.

A qualified psychiatrist conducted a face-to-face clinical interview and mental-status examination guided by the structured study case-report form. The focused PAD assessment systematically covered symptom onset and context, triggers, anticipatory anxiety, somatic symptoms, avoidance, insight, functional impairment, psychiatric history, comorbid symptoms, and alternative explanations. The psychiatrist integrated interview and clinical information to determine PAD diagnoses. No separately validated structured diagnostic interview was documented in the study archive, and assessor identifiers were not retained; formal inter-rater diagnostic reliability therefore could not be estimated retrospectively.

The Hamilton Anxiety Rating Scale was available for supportive clinical characterization when indicated but did not define PADs and was not included in the analyses [16]. Among participants diagnosed with PADs, phobic symptoms were characterized descriptively with the 15-item Fear Questionnaire (FQ), which covers agoraphobic, social-phobic, and specific-phobia situations [17,18]. FQ scores did not determine diagnostic case status. The study archive did not document formal cross-cultural validation of the Vietnamese FQ version used; accordingly, FQ scores are interpreted descriptively.

### 2.4. Outcome ascertainment

The primary outcome was the presence of any PAD. A qualified psychiatrist made diagnoses according to ICD-10 clinical criteria [19]; questionnaire scores alone did not determine case status. ICD-10 was the prespecified diagnostic framework in the study protocol and clinical setting, and DSM criteria were not applied in parallel. The assessment considered the focus and proportionality of fear, anticipatory anxiety, avoidance, functional impairment, insight, duration, and alternative explanations.

PADs were classified as agoraphobia, social phobia, or specific phobia, and subtypes could co-occur. Social phobia required fear or avoidance of social situations involving possible scrutiny. Specific phobia required fear or avoidance restricted to a circumscribed object or situation not better classified as agoraphobia or social phobia. Agoraphobia involved fear or avoidance of situations in which escape or access to help might be difficult.

### 2.5. Variables

Potential associated factors were identified a priori within a biopsychosocial framework. Demographic and socioeconomic variables included age, sex, education, occupation, marital status, economic status, and place of residence.

History variables included family history of physical or psychiatric illness, personal physical comorbidity, prior psychiatric history, psychological trauma, psychoactive-substance use, and concurrent anxiety/depressive symptoms recorded during psychiatric assessment. The concurrent symptom indicator was analytically distinct from prior psychiatric history and from the exclusion criterion for a psychiatric condition severe enough to preclude valid assessment.

Dermatological variables included acne onset and duration, lesion type and distribution, acne severity, scarring and other sequelae, treatment history, corticosteroid and isotretinoin use, follow-up frequency, treatment changes, and treatment response. For regression, severe acne was contrasted with mild/moderate acne. Treatment response was analyzed as poor (partial or no response) versus marked response in the primary model.

Acne-related life impact was recorded using a study-specific three-level case-report-form item (little, moderate, or major impact); no validated acne-specific or dermatology-specific quality-of-life questionnaire was administered. Major impact was contrasted with little/moderate impact in the primary model. Study-specific psychosocial items included emotional stability/anxiety-proneness, introversion/extraversion, coping tendency, and impulsivity/cautiousness. Anxiety-proneness was recorded as stable/carefree, fairly stable, or easily anxious; the primary model contrasted ‘easily anxious’ with the other categories. Avoidant coping was recorded as approach versus avoidance. These items were not validated psychometric scales, and study-specific validity or inter-rater reliability estimates were not available. Self-esteem, body-image concern, social inhibition, and perceived stigma were not treated as independently measured variables because the analytic archive did not contain corresponding validated measures; appearance-related stigma/isolation, where recorded, formed part of the psychological-trauma history rather than a separate stigma scale. S4 Table summarizes the operational definitions and coding.

### 2.6. Statistical analysis

Data were checked, cleaned, and analyzed with IBM SPSS Statistics. Data-entry checks included repeat review and double-entry verification when required.

Continuous data were described by mean (SD) or median (IQR), according to distribution, and categorical data by counts and percentages. The proportion with PADs was calculated among all included participants, and its 95% CI was estimated with the Wilson score method [20]. Subtype-specific proportions used the total sample as the denominator; distributions among PAD cases used the number with any PAD as the denominator.

In univariable analyses, categorical variables were compared with the χ² test or Fisher’s exact test when expected counts were small. Continuous variables were compared with an independent-samples t test when approximately normally distributed and with the Mann–Whitney U test otherwise. Crude odds ratios (ORs) and 95% CIs were estimated with univariable logistic regression.

The reported multivariable logistic regression model included nine covariates selected for clinical relevance within the biopsychosocial framework and constrained by the number of PAD events: psychological trauma, study-specific anxiety-proneness rating, physical comorbidity, concurrent anxiety/depressive symptoms, severe acne, acne scarring, current isotretinoin use, major acne-related life impact, and poor treatment response. Age and sex were evaluated in univariable analyses but were not included in the reported model.

With 69 PAD events and nine covariates (7.7 events per modeled covariate), the model was considered exploratory and potentially vulnerable to overfitting and imprecision. Although the conventional 10-events-per-variable rule is not an absolute threshold [21], wide CIs and sparse categories warranted caution. The analysis plan allowed Firth penalized logistic regression when sparse cells, quasi-complete separation, or unstable maximum-likelihood estimates were encountered [22,23]. Model discrimination was summarized by the area under the receiver operating characteristic curve (AUC), explanatory fit by McFadden’s pseudo-R^2^, and calibration by the Hosmer–Lemeshow statistic.

Two sensitivity analyses were performed. The first repeated the model after excluding participants with concurrent anxiety/depressive symptoms. The second replaced the binary poor-response variable with a three-level treatment-response variable (marked, partial, or none) to assess the robustness of the coding choice. Because the latter analysis yielded a very large and imprecise estimate for no response, it was interpreted as evidence of sparse data and possible separation rather than as a precise effect estimate.

Missingness was tabulated before analysis. The PAD outcome and all covariates in the primary model were complete, so no imputation was performed. FQ domain scores were available for all 69 PAD cases, whereas the reported FQ total score was available for 67. S1 Table summarizes completeness.

All tests were two-sided, and p < 0.05 was used as a descriptive threshold. No adjustment was made for multiple comparisons. Because the analyses were exploratory, interpretation emphasized effect estimates, 95% CIs, precision, and clinical plausibility rather than statistical significance alone.

### 2.7. Ethics

The Ethics Committee for Biomedical Research of the National Hospital of Dermatology and Venereology, Vietnam, approved the study before recruitment (approval No. 131/HĐĐĐ-BVDLTW; 27 November 2025).

## 3. Results

### 3.1. Participant characteristics

The primary analysis included 280 participants with acne vulgaris. The mean age was 21.3 years (SD, 4.3), and 192 (68.6%) were women. Severe acne was recorded in 143 (51.1%), acne scarring in 162 (57.9%), current isotretinoin use in 174 (62.1%), and poor treatment response in 161 (57.5%) (Table 1).

**Table 1:** Participant characteristics according to phobic anxiety disorder status among patients with acne vulgaris.

Characteristic	Total, n = 280	No PADs, n = 211	PADs, n = 69	p value
Age, years, mean ± SD	21.3 ± 4.3	21.3 ± 4.3	21.3 ± 4.5	0.973
Age ≥ 25 years	48 (17.1)	35 (16.6)	13 (18.8)	0.667
Female sex	192 (68.6)	143 (67.8)	49 (71.0)	0.615
Education ≥ upper secondary	265 (94.6)	199 (94.3)	66 (95.7)	0.669
Urban residence	165 (58.9)	127 (60.2)	38 (55.1)	0.454
Age at acne onset, years, mean ± SD	15.5 ± 3.0	15.4 ± 2.8	15.8 ± 3.5	0.345
Acne duration ≥3 years	226 (80.7)	171 (81.0)	55 (79.7)	0.876
Severe acne	143 (51.1)	99 (46.9)	44 (63.8)	0.016
Acne scarring	162 (57.9)	115 (54.5)	47 (68.1)	0.048
Current isotretinoin use	174 (62.1)	129 (61.1)	45 (65.2)	0.544
Treatment changed in previous 12 months	44 (15.7)	18 (8.5)	26 (37.7)	<0.001
Poor treatment response	161 (57.5)	102 (48.3)	59 (85.5)	<0.001
Major acne-related life impact	155 (55.4)	106 (50.2)	49 (71.0)	0.003
Physical comorbidity	78 (27.9)	38 (18.0)	40 (58.0)	<0.001
Concurrent anxiety/depressive symptoms	43 (15.4)	26 (12.3)	17 (24.6)	0.016
Study-specific anxiety-proneness rating	48 (17.1)	18 (8.5)	30 (43.5)	<0.001
Psychological trauma	10 (3.6)	4 (1.9)	6 (8.7)	0.016

*Note:* Values are n (%) unless otherwise indicated. P values compare participants with and without PADs using the χ² or Fisher’s exact test for categorical variables and the independent-samples t test for age variables. PADs, phobic anxiety disorders; SD, standard deviation.

### 3.2. Prevalence and clinical phenotype of PADs

Sixty-nine of 280 participants met ICD-10 criteria for at least one PAD (24.6%; 95% CI, 20.0%–30.0%). Social phobia affected 64 participants (22.9% of the total sample; 92.8% of PAD cases), specific phobia affected 26 (9.3%; 37.7%), and agoraphobia affected one (0.4%; 1.4%) (Table 2).

**Table 2:** Prevalence and clinical subtypes of phobic anxiety disorders among patients with acne vulgaris.

PADs phenotype	n/N		%
Any PADs	69/280		24.6
95% CI for PADs prevalence	—		20.0–30.0
**A. Subtype-specific prevalence**			
**Subtype**	**n**	**% of total sample**	**% among PADs cases**
Social phobia	64	22.9	92.8
Specific phobia	26	9.3	37.7
Agoraphobia	1	0.4	1.4
**B. Subtype overlap among participants with phobic anxiety disorders**			
**Number of PADs subtypes**	**n = 69**		**% among PADs cases**
One subtype	47		68.1
Two subtypes	22		31.9
Three subtypes	0		0.0

PAD subtypes were not mutually exclusive. Forty-seven of 69 PAD cases (68.1%) had one subtype and 22 (31.9%) had two; none had all three subtypes.

### 3.3. Fear Questionnaire profile and phobic symptom burden

**Fig 1:**
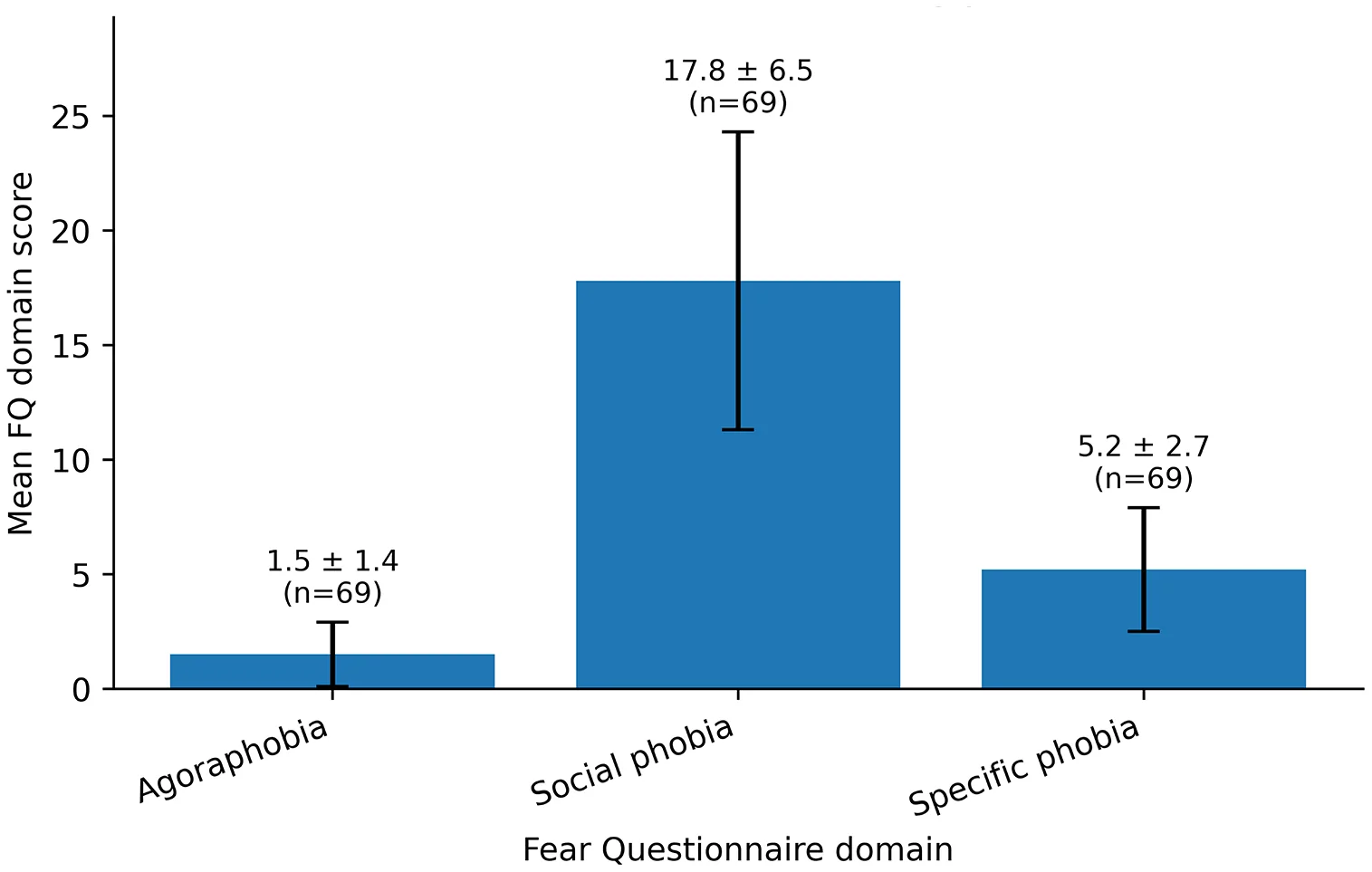
Fear Questionnaire domain scores among patients with acne vulgaris and phobic anxiety disorders. Bars show means and error bars show 95% confidence intervals calculated from the reported standard deviations; domain scores were available for all 69 PAD cases. FQ, Fear Questionnaire; PADs, phobic anxiety disorders.

Among participants with PADs, the social-phobia domain had the highest mean FQ score, followed by the specific-phobia and agoraphobia domains (Fig 1).

Table 3 presents the FQ score distributions. Among PAD cases, the mean social-phobia domain score was 17.8 (SD, 6.5), compared with 5.2 (SD, 2.7) for specific phobia and 1.5 (SD, 1.4) for agoraphobia. The mean total score among the 67 cases with an available total was 24.3 (SD, 6.4).

**Table 3:** Fear Questionnaire scores among patients with acne vulgaris and phobic anxiety disorders.

FQ measure	n	Mean ± SD	Median (IQR)	Range
Total FQ score	67	24.3 ± 6.4	25.0 (20.0–30.0)	11–37
Agoraphobia domain	69	1.5 ± 1.4	1.0 (1.0–2.0)	0–10
Social phobia domain	69	17.8 ± 6.5	18.0 (14.0–23.0)	5–30
Specific phobia domain	69	5.2 ± 2.7	4.0 (3.0–7.0)	2–14

### 3.4. Dermatological and psychosocial correlates of PADs

In univariable analyses, age, sex, education, residence, age at acne onset, acne duration, acne distribution, and current isotretinoin use had estimates close to the null with CIs that included 1.00 (Table 4).

**Table 4:** Univariable associations with phobic anxiety disorders among patients with acne vulgaris.

Factor	PADs prevalence in exposed group	PADs prevalence in reference group	Crude OR (95% CI)	p value
Age ≥ 25 years	13/48 (27.1)	56/232 (24.1)	1.17 (0.58–2.36)	0.667
Female sex	49/192 (25.5)	20/88 (22.7)	1.17 (0.64–2.11)	0.615
Education ≥ upper secondary	66/265 (24.9)	3/15 (20.0)	1.33 (0.36–4.85)	0.669
Psychological trauma	6/10 (60.0)	63/270 (23.3)	4.93 (1.35–18.02)	0.016
Study-specific anxiety-proneness rating	30/48 (62.5)	39/232 (16.8)	8.25 (4.19–16.25)	<0.001
Physical comorbidity	40/78 (51.3)	29/202 (14.4)	6.28 (3.47–11.36)	<0.001
Concurrent anxiety/depressive symptoms	17/43 (39.5)	52/237 (21.9)	2.33 (1.17–4.61)	0.016
Acne onset >15 years	30/115 (26.1)	39/165 (23.6)	1.14 (0.66–1.98)	0.640
Acne duration >5 years	28/128 (21.9)	41/152 (27.0)	0.76 (0.44–1.32)	0.325
Multiple-site acne	25/92 (27.2)	44/188 (23.4)	1.22 (0.69–2.16)	0.492
Severe acne	44/143 (30.8)	25/137 (18.2)	1.99 (1.14–3.49)	0.016
Acne scarring	47/162 (29.0)	22/118 (18.6)	1.78 (1.00–3.17)	0.048
Current isotretinoin use	45/174 (25.9)	24/106 (22.6)	1.19 (0.68–2.10)	0.544
Treatment change	26/44 (59.1)	43/236 (18.2)	6.48 (3.27–12.87)	<0.001
Regular prior treatment	31/88 (35.2)	38/192 (19.8)	2.20 (1.25–3.87)	0.006
Poor treatment response	59/161 (36.6)	10/119 (8.4)	6.30 (3.06–12.99)	<0.001
Major acne-related life impact	49/155 (31.6)	20/125 (16.0)	2.43 (1.35–4.36)	0.003

*Note:* Reference groups are the absence of the listed characteristic unless an explicit comparator is shown. PADs, phobic anxiety disorders; OR, odds ratio; CI, confidence interval.

The largest crude associations were observed for study-specific anxiety-proneness rating (OR, 8.25; 95% CI, 4.19–16.25), treatment change (OR, 6.48; 95% CI, 3.27–12.87), physical comorbidity (OR, 6.28; 95% CI, 3.47–11.36), and poor treatment response (OR, 6.30; 95% CI, 3.06–12.99). Severe acne, scarring, concurrent anxiety/depressive symptoms, regular prior treatment, and major acne-related life impact also had crude CIs that excluded 1.00.

### 3.5. Multivariable model for PADs

**Fig 2:**
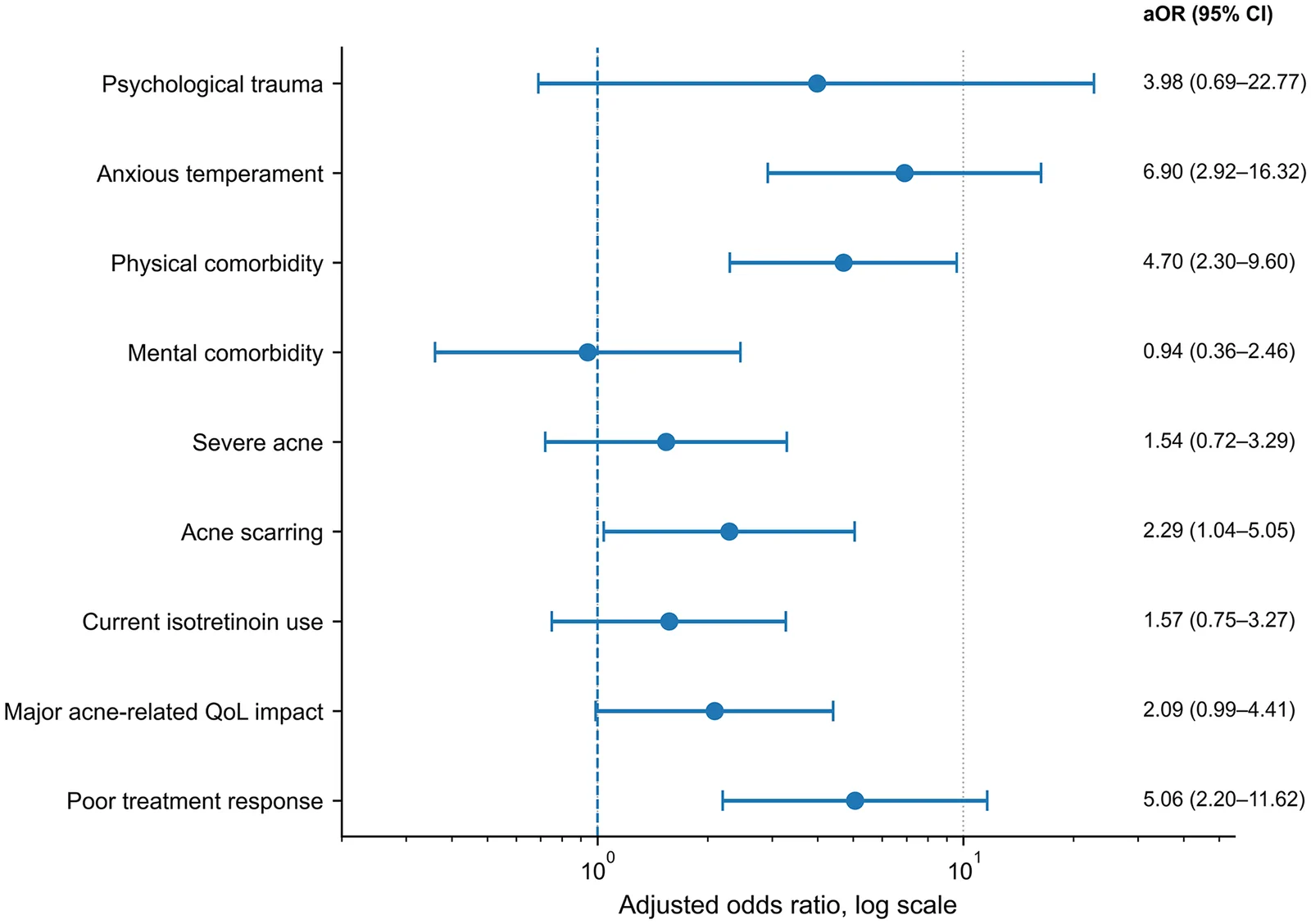
Adjusted associations with phobic anxiety disorders among patients with acne vulgaris. Points show adjusted odds ratios and horizontal lines show 95% confidence intervals; the dashed vertical line marks an odds ratio of 1. The model included 280 participants and 69 PAD events. Exact estimates are reported in Table 5. PADs, phobic anxiety disorders.

The exploratory multivariable model included the nine specified covariates (Fig 2 and Table 5). Study-specific anxiety-proneness rating, physical comorbidity, acne scarring, and poor treatment response had adjusted 95% CIs that excluded 1.00.

**Table 5:** Multivariable associations with phobic anxiety disorders among patients with acne vulgaris.

Factor	Adjusted OR	95% CI	p value
Psychological trauma	3.98	0.69–22.77	0.121
Study-specific anxiety-proneness rating	6.90	2.92–16.32	<0.001
Physical comorbidity	4.70	2.30–9.60	<0.001
Concurrent anxiety/depressive symptoms	0.94	0.36–2.46	0.907
Severe acne	1.54	0.72–3.29	0.268
Acne scarring	2.29	1.04–5.05	0.040
Current isotretinoin use	1.57	0.75–3.27	0.231
Major acne-related life impact	2.09	0.99–4.41	0.052
Poor treatment response	5.06	2.20–11.62	<0.001

*Note:* N = 280; PAD events = 69; nine covariates (7.7 events per modeled covariate). Model performance: AUC = 0.856; McFadden’s pseudo-R^2^ = 0.326; Hosmer–Lemeshow p = 0.383. Estimates are exploratory and should be interpreted with attention to CI width and potential overfitting. OR, odds ratio; CI, confidence interval; AUC, area under the receiver operating characteristic curve.

Study-specific anxiety-proneness rating had the largest adjusted association (aOR, 6.90; 95% CI, 2.92–16.32), followed by poor treatment response (aOR, 5.06; 95% CI, 2.20–11.62), physical comorbidity (aOR, 4.70; 95% CI, 2.30–9.60), and acne scarring (aOR, 2.29; 95% CI, 1.04–5.05). Because anxiety-proneness was not measured with a validated temperament instrument, its estimate should be interpreted as exploratory.

Adjusted estimates for psychological trauma, concurrent anxiety/depressive symptoms, severe acne, current isotretinoin use, and major acne-related life impact were imprecise and had 95% CIs that included 1.00. The estimate for major acne-related life impact was 2.09 (95% CI, 0.99–4.41), indicating substantial uncertainty around a potentially relevant association.

The model AUC was 0.856, McFadden’s pseudo-R^2^ was 0.326, and the Hosmer–Lemeshow p value was 0.383.

### 3.6. Sensitivity analyses

The PAD outcome and all covariates in the primary model were complete. FQ domain scores were available for all PAD cases, whereas the FQ total was unavailable for two cases (S1 Table).

After participants with concurrent anxiety/depressive symptoms were excluded (N = 237; 52 PAD events), adjusted associations remained for study-specific anxiety-proneness rating, physical comorbidity, and poor treatment response. The estimate for acne scarring was 2.47 (95% CI, 0.98–6.19) (S2 Table).

When treatment response was modeled in three levels, the estimate for no response versus marked response was very large and imprecise (aOR, 137.92; 95% CI, 29.35–648.19), consistent with sparse data or near-separation. The estimate for partial versus marked response was 2.05 (95% CI, 0.82–5.16) (S3 Table). These estimates should not be interpreted as precise effect sizes.

Sensitivity analyses yielded similar directions of association for anxiety-proneness, physical comorbidity, and poor treatment response; after sample restriction, the estimate for scarring became less precise.

## 4. Discussion

In this cross-sectional convenience sample of 280 acne outpatients at a national dermatology hospital in Vietnam, 24.6% met ICD-10 criteria for at least one PAD, and social phobia predominated. The exploratory adjusted model identified associations with study-specific anxiety-proneness rating, physical comorbidity, acne scarring, and poor treatment response, whereas clinician-rated severe acne was not independently associated. These findings characterize a tertiary-care sample and should not be interpreted as population prevalence, prediction, or causal effects.

The observed PAD burden is compatible with broader evidence linking acne with anxiety, depression, psychosocial distress, and impaired quality of life [2,4–6,11,12,24]. However, most prior acne studies used symptom scales, whereas the present study used psychiatrist-led ICD-10 case ascertainment; differences in sampling, instruments, age mix, and clinical setting preclude direct numerical comparison [4–6,10–12].

Social phobia accounted for most PAD diagnoses, consistent with literature linking visible acne with social-evaluative concern and appearance-related stigma [9–12]. The FQ social-phobia domain was highest among PAD cases, providing descriptive concordance with the clinical phenotype. Because perceived stigma was not independently measured and the design is cross-sectional, we cannot determine whether acne-related scrutiny contributed to PAD onset or whether pre-existing social anxiety shaped treatment-seeking and symptom interpretation.

Scarring remained associated with PADs after adjustment, whereas severe acne did not. Scars can remain visible after inflammatory lesions improve, and current acne guidance recognizes scarring and psychosocial burden as clinically important [1,11–13]. The contrast between scarring and severe acne may indicate that visible sequelae or accumulated disease experience matter beyond current inflammatory severity, but residual confounding, measurement error in the three-level severity rating, and reverse causation remain possible.

Poor treatment response was also strongly associated with PADs. Repeated or unsatisfactory treatment can coexist with prolonged disease burden, frustration, and appearance-related distress [11,12]. Conversely, phobic avoidance may affect attendance, adherence, or perceptions of treatment success. Importantly, treatment response in this study was a study-specific clinical classification rather than a standardized longitudinal lesion-count or IGA-change endpoint, so the estimate should not be interpreted as the effect of objectively measured treatment failure.

The study-specific anxiety-proneness rating had the largest adjusted association. Because this item was derived from a categorical case-report-form assessment of emotional stability/anxiety-proneness rather than a validated temperament instrument, construct overlap with PAD symptoms and common-method bias may inflate the estimate. The association should therefore be interpreted as an exploratory clinical signal, not as evidence that a validated temperament trait predicts PADs. Physical comorbidity also remained associated, supporting the relevance of broader health burden but without establishing mechanism.

The estimate for major acne-related life impact was imprecise and its CI included 1.00. This variable was a three-level study-specific rating rather than a validated acne-specific quality-of-life instrument. Psychodermatology literature nevertheless supports assessing psychosocial burden alongside cutaneous severity [11,12]. Future work should use validated acne-specific or dermatology-specific quality-of-life measures and prespecified longitudinal assessments.

Selection and spectrum bias are important. The study was conducted at a single national tertiary referral hospital; 51.1% of participants had severe acne, 62.1% were receiving isotretinoin, and 57.5% had poor treatment response. This treatment-experienced case mix may carry greater dermatological and psychosocial burden than community or primary-care acne populations. Statistical adjustment for severity and isotretinoin cannot remove referral selection, so the 24.6% proportion should not be generalized as a national prevalence estimate.

For clinical practice, the findings support attention to avoidance, fear of scrutiny, and interference with school, work, or relationships when patients with acne report persistent distress, scarring, or difficult treatment courses [11,12]. Brief validated social-anxiety screeners such as the Social Phobia Inventory (SPIN) or Mini-SPIN may be considered for case finding in appropriately validated settings [25,26], but a positive screen is not a diagnosis. The present study did not validate a Vietnamese screening threshold or referral algorithm; screening should therefore support, not replace, clinical assessment and referral when indicated.

Strengths include psychiatrist-led ICD-10 case ascertainment, characterization of PAD subtypes alongside FQ profiles, consideration of dermatological, treatment-related, somatic, and psychosocial factors, and complete primary outcome and model covariate data. Sensitivity analyses also showed that key associations were directionally similar after excluding participants with concurrent anxiety/depressive symptoms and after alternative treatment-response coding, although precision varied.

Several limitations affect inference. Cross-sectional data preclude temporal and causal conclusions. Recruitment used convenience sampling, and the archived records did not permit reconstruction of the numbers approached, refusing participation, or excluded before enrollment, preventing a complete response rate or participant-flow diagram. The primary model contained 69 PAD events and nine covariates (7.7 events per modeled covariate), creating a risk of overfitting and unstable estimates; the wide CIs and near-separation in the three-level treatment-response sensitivity analysis reinforce this concern [21]. No multiplicity adjustment was applied, so the regression results should be considered exploratory.

Measurement limitations are also important. Acne severity, treatment response, acne-related life impact, and anxiety-proneness used study-specific clinical/case-report-form classifications; no validation or inter-rater reliability estimates were available for these measures. The analytic archive did not document a separately validated structured psychiatric interview, assessor identifiers, or formal cross-cultural validation of the Vietnamese FQ; FQ scores were therefore used descriptively rather than diagnostically. Concurrent anxiety/depressive symptoms could overlap with PAD manifestations, and assessor non-blinding may contribute to common-method or classification bias. Residual confounding and reverse causation remain possible. Multicentre prospective studies using standardized psychiatric, psychosocial, acne-severity, treatment-response, and quality-of-life measures are needed.

## 5. Conclusion

Clinician-diagnosed PADs—especially social phobia—were a clinically relevant feature of this tertiary-care acne sample. The findings support attention to social avoidance and psychological burden in acne care, while the referral setting, exploratory model, and study-specific measures require confirmation in prospective multicentre studies using standardized assessments.

## Supporting information

S1 Data Dataset. (SAV)

S1 Table Completeness of data used in the primary and descriptive analyses among patients with acne vulgaris. (DOCX)

S2 Table Sensitivity analysis excluding participants with concurrent anxiety/depressive symptoms among patients with acne vulgaris. (DOCX)

S3 Table Sensitivity analysis using three-level treatment-response coding among patients with acne vulgaris. (DOCX)

S4 Table Operational definitions and coding of selected study variables among patients with acne vulgaris. (DOCX)
