# Supplementary material for: Phobic anxiety disorders among patients with acne vulgaris at a national dermatology hospital in Vietnam: Prevalence, clinical phenotype, and associated factors
Source: PLoS One. 2026 Sep 10;21(9):e0357803. doi: 10.1371/journal.pone.0357803 (PMC13561362; doi:10.1371/journal.pone.0357803)
Supplement: Supplementary file 1 [file pone.0357803.s002.docx]

**S1 Table. Completeness of data used in the primary and descriptive analyses among patients with acne vulgaris**

| **Variable group** | **Missing data** |
| --- | --- |
| Primary PAD outcome | 0/280 |
| Demographic variables in main model | 0/280 |
| Dermatological variables in main model | 0/280 |
| Psychosocial/history variables in main model | 0/280 |
| FQ domain scores among PAD cases | 0/69 |
| FQ total score among PAD cases | 2/69 |
