# Supplementary material for: Phobic anxiety disorders among patients with acne vulgaris at a national dermatology hospital in Vietnam: Prevalence, clinical phenotype, and associated factors
Source: PLoS One. 2026 Sep 10;21(9):e0357803. doi: 10.1371/journal.pone.0357803 (PMC13561362; doi:10.1371/journal.pone.0357803)
Supplement: Supplementary file 2 [file pone.0357803.s003.docx]

**S2 Table. Sensitivity analysis excluding participants with concurrent anxiety/depressive symptoms among patients with acne vulgaris**

| **Factor** | **Adjusted odds ratio** | **95% CI** | **P value** |
| --- | --- | --- | --- |
| Psychological trauma | 3.13 | 0.48–20.30 | 0.231 |
| Study-specific anxiety-proneness rating | 12.48 | 4.15–37.49 | <0.001 |
| Physical comorbidity | 6.06 | 2.68–13.67 | <0.001 |
| Severe acne | 1.81 | 0.74–4.42 | 0.191 |
| Acne scarring | 2.47 | 0.98–6.19 | 0.054 |
| Current isotretinoin use | 1.23 | 0.53–2.84 | 0.628 |
| Major acne-related life impact | 1.51 | 0.65–3.51 | 0.336 |
| Poor treatment response | 6.13 | 2.29–16.38 | <0.001 |

*Note:* N=237; PAD events=52; AUC=0.872. Concurrent anxiety/depressive symptoms were omitted because participants with that characteristic were excluded. OR, odds ratio; CI, confidence interval; AUC, area under the receiver operating characteristic curve.
