# Supplementary material for: Phobic anxiety disorders among patients with acne vulgaris at a national dermatology hospital in Vietnam: Prevalence, clinical phenotype, and associated factors
Source: PLoS One. 2026 Sep 10;21(9):e0357803. doi: 10.1371/journal.pone.0357803 (PMC13561362; doi:10.1371/journal.pone.0357803)
Supplement: Supplementary file 3 [file pone.0357803.s004.docx]

**S3 Table. Sensitivity analysis using three-level treatment-response coding among patients with acne vulgaris**

| **Factor** | **Adjusted odds ratio** | **95% CI** | **P value** |
| --- | --- | --- | --- |
| No treatment response vs marked response | 137.92 | 29.35–648.19 | <0.001 |
| Partial response vs marked response | 2.05 | 0.82–5.16 | 0.127 |

*Note:* The model used the same covariate structure as the primary multivariable model, except that binary poor treatment response was replaced by a three-level variable (marked, partial, or no response). Only treatment-response coefficients are displayed. The very large and imprecise estimate for no response is consistent with sparse data or near-separation and should not be interpreted as a precise effect size. OR, odds ratio; CI, confidence interval. The primary model contained 69 PAD events and nine covariates (7.7 events per modeled covariate), so estimates are interpreted as exploratory.
