# Supplementary material for: Phobic anxiety disorders among patients with acne vulgaris at a national dermatology hospital in Vietnam: Prevalence, clinical phenotype, and associated factors
Source: PLoS One. 2026 Sep 10;21(9):e0357803. doi: 10.1371/journal.pone.0357803 (PMC13561362; doi:10.1371/journal.pone.0357803)
Supplement: Supplementary file 4 [file pone.0357803.s005.docx]

**S4 Table. Operational definitions and coding of selected study variables among patients with acne vulgaris**

| **Variable** | **Operational definition and analytic coding** | **Measurement status / interpretation** |
| --- | --- | --- |
| Study-specific anxiety-proneness rating | Case-report-form item on emotional stability/anxiety-proneness with categories stable/carefree, fairly stable, and easily anxious. Primary model: easily anxious vs the other categories. | Study-specific categorical item; not a validated temperament scale. No study-specific validity or inter-rater reliability estimate was available. |
| Avoidant coping | Case-report-form coping item recorded as approach vs avoidance. | Study-specific categorical item; not a validated coping questionnaire. |
| Acne-related life impact | Case-report-form item recorded as little, moderate, or major impact. Primary model: major vs little/moderate. | No validated acne-specific or dermatology-specific quality-of-life questionnaire was administered for this variable. |
| Treatment response | Recorded on the dermatology case-report form at the study visit as marked, partial, or no response. Primary model: poor response (partial/no) vs marked response. | Pragmatic study-specific clinical classification; not a standardized longitudinal lesion-count reduction, numeric IGA-change endpoint, or validated patient-satisfaction scale. |
| Acne severity | Three-level clinician global classification: mild, moderate, or severe. Primary model: severe vs mild/moderate. | Informed by Investigator's Global Assessment principles; the analytic archive did not contain a formal numerical IGA score. |
| Concurrent anxiety/depressive symptoms | Binary indicator recorded during psychiatric assessment for concurrent anxiety and/or depressive symptoms among included participants. | Distinct from prior psychiatric history and from the exclusion criterion for a psychiatric condition severe enough to preclude valid assessment. |
| Psychological trauma | History item recorded in the structured case-report form; appearance-related stigma/isolation, where recorded, was a possible trauma-history category rather than a separate stigma measure. | No independent validated perceived-stigma scale was administered. |
| Fear Questionnaire | 15-item FQ used descriptively among participants diagnosed with PADs; domain scores were available for 69/69 PAD cases and the total score for 67/69. | Validated instrument in the original literature [1, 2], but the study archive did not document formal cross-cultural validation of the Vietnamese version used. FQ scores did not determine PAD diagnosis. |

*Note:* IGA, Investigator's Global Assessment; FQ, Fear Questionnaire; PADs, phobic anxiety disorders. The table distinguishes standardized instruments from study-specific clinical/case-report-form classifications and summarizes the coding used in the reported analyses.

**References**

[1]	I. M. Marks and A. M. Mathews, "Brief standard self-rating for phobic patients," *Behaviour Research and Therapy,* vol. 17, no. 3, pp. 263-267, 1979/01/01/ 1979, doi: <https://doi.org/10.1016/0005-7967(79)90041-X>.

[2]	F. J. Van Zuuren, "The Fear Questionnaire: Some Data on Validity, Reliability and Layout," *British Journal of Psychiatry,* vol. 153, no. 5, pp. 659-662, 1988, doi: 10.1192/bjp.153.5.659.
