# Supplementary material for: Phobic anxiety disorders among patients with acne vulgaris at a national dermatology hospital in Vietnam: Prevalence, clinical phenotype, and associated factors
Source: PLoS One. 2026 Sep 10;21(9):e0357803. doi: 10.1371/journal.pone.0357803 (PMC13561362; doi:10.1371/journal.pone.0357803)
Supplement: Supplementary file 5 [file pone.0357803.s007.docx]

**Dear Reviewer #1,**

Thank you for your thoughtful and constructive comments. We have carefully reviewed and revised our manuscript accordingly. We have also highlighted the changes in yellow for your convenience. Here are the details:

**Comment 1: The psychosocial variables are poorly defined (anxious temperament, anxious temperament, emotional instability, social inhibition, body-image concern, avoidant coping, self-esteem, perceived stigma). How were these variables assessed? Were validated instruments used, if yes, which and please describe for the dermatological readership. Or were they rated as clinical impressions? Please provide evidence of validity and reliability. Especially for the variables which gave strong association (anxious temperament, etc).**

**Response 1:** We agree with you that the previous description was insufficient and could incorrectly imply that all psychosocial constructs were measured using validated psychometric instruments.

We have therefore substantially revised the Methods and terminology. The available study data show that these characteristics were recorded as study-specific categorical items in the structured case-report form and face-to-face clinical assessment rather than as separate validated psychometric scales.

In particular, the variable previously termed “anxious temperament” was derived from a case-report-form item concerning emotional stability/anxiety proneness. Participants categorized as “easily anxious” were contrasted with those categorized as stable/carefree or relatively stable. Avoidant coping was similarly recorded as a categorical coping tendency.

The source dataset does not contain independent validated scales for self-esteem, body-image concern, social inhibition, or perceived stigma. We have therefore removed wording suggesting that these were independently measured psychosocial constructs.

Importantly, no study-specific psychometric validation or inter-rater reliability evaluation was performed for these categorical clinical items. We now state this explicitly as a limitation. We have also revised the Discussion to emphasize that the strong association observed for anxiety proneness should be interpreted cautiously because of potential construct overlap with PAD symptoms and possible common-method bias.

**Comment 2: ‘Poor treatment response’ is another strong predictor. But what exactly is treatment response? Who evaluated it? Dermatologist? Patient? Both? Objective improvement? IGA? Patient satisfaction? This variable needs a much clearer operational definition.**

**Response 2:** We agree and have clarified this variable substantially.

Treatment response was recorded in the structured study case-report form using three categories: no response, partial response, and marked response. For the primary regression analysis, no response and partial response were combined as “poor treatment response” and compared with marked response.

This variable was recorded as part of the clinical assessment, treatment history, and medical-record review. The available analytic data do not identify a separate assessor-specific field that would allow us to classify the measure retrospectively as exclusively dermatologist-rated or patient-rated.

Importantly, treatment response was not defined using a standardized longitudinal Investigator’s Global Assessment change, percentage reduction in lesion count, or a validated patient-satisfaction instrument. We have revised the Methods accordingly and now describe it explicitly as a study-specific clinical treatment-response classification.

We have also strengthened the Discussion and limitations to acknowledge possible measurement error, subjective components of treatment-response assessment, and the inability of the cross-sectional design to establish whether poor response contributed to PADs or whether psychological factors influenced treatment experience, adherence, or reporting.

**Comment 3: Please comment on Selection bias. The study is based on one tertiary referral hospital. What about generalizability?**

**Response 3:** We agree and have considerably expanded the discussion of selection and spectrum bias.

The study used convenience sampling at a single national tertiary dermatology hospital. The sample consequently contained a substantial proportion of patients with clinically complex acne: 51.1% had severe acne, 62.1% were receiving isotretinoin, and 57.5% had poor treatment response.

These characteristics may reflect referral of patients with more severe, persistent, or treatment-experienced disease and could contribute to a higher psychological burden than would be expected in community or primary-care acne populations.

We therefore now explicitly state that the observed 24.6% proportion of PADs is a hospital-based sample proportion and should not be interpreted as a national or population prevalence estimate.

We have also added that adjustment for measured clinical characteristics cannot eliminate referral-selection or spectrum bias. Multicentre studies incorporating primary dermatology, general clinical, and community settings are needed to assess external validity.

**Comment 4: 9 variables were evaluated for 69 events. It seems there might be too many variables. CIs are quite unstable. Please mention this in more detail as a limitation.**

**Response 4:** We agree.

The primary multivariable model contained nine covariates and 69 PAD events, corresponding to approximately 7.7 events per modeled covariate. We now report this explicitly and describe the multivariable analysis as exploratory.

We have expanded the limitations to acknowledge the risk of overfitting, coefficient instability, and reduced precision associated with the modest event count. Interpretation now emphasizes effect estimates and 95% confidence intervals rather than statistical significance alone.

The sensitivity analyses are retained to evaluate the robustness of the main findings, but we explicitly state that they do not eliminate the potential for model instability. In particular, the very large estimate observed when treatment response was modeled in three levels is now clearly described as evidence of sparse data/near-separation and not as a precise effect estimate.

**Comment 5: STROBE recommends a flow diagram of participants. How many were approached? How many refused? How many were excluded and for what reason?**

**Response 5:** We agree that complete recruitment-flow information would strengthen reporting.

The available analytic dataset contains the 280 participants who completed the PAD assessment. However, a separate screening log documenting the total number approached, refusing participation, or excluded at each stage was not retained in the archived study files available for the present analysis.

We therefore cannot retrospectively reconstruct these numbers accurately without introducing unverifiable information. For this reason, we have not generated a numerical flow diagram containing estimated or inferred recruitment counts.

Instead, we have clarified the convenience-sampling procedure in the Methods and added incomplete reporting of recruitment flow and response rate as a limitation.

**Comment 6: Severity is said to use an IGA-oriented approach instead of a validated grading system. IGA is not given in full. Was this the Investigator's Global Assessment?**

**Response 6:** Yes. We thank you for identifying this ambiguity.

We have now defined IGA in full as Investigator’s Global Assessment.

We have also clarified that the analytic dataset contains a three-category clinician global acne severity classification—mild, moderate, or severe—rather than a formal numerical validated IGA score.

The Methods have therefore been revised to avoid implying that a fully standardized numerical IGA instrument was prospectively recorded.

**Comment 7: Why was ICD-10 chosen instead of DSM?**

**Response 7:** We have now added this clarification to the Methods.

ICD-10 was the prespecified diagnostic framework used in the study protocol for psychiatric assessment. PAD diagnoses were based on psychiatrist-led clinical assessment against ICD-10 clinical criteria rather than questionnaire thresholds alone.

Because the study protocol was designed around ICD-10 diagnostic categories, DSM criteria were not applied retrospectively as a parallel diagnostic system. We have clarified this rationale in the revised manuscript.

**Comment 8: What is severe psychiatric disorder? And you include mental comorbidity as a covariate. Please elaborate.**

**Response 8:** We agree that the previous wording did not adequately distinguish these concepts.

The exclusion criterion referred to a previously diagnosed psychiatric disorder of sufficient current severity to substantially interfere with valid participation in the clinical interview and study assessment. It was not intended to exclude all participants with any psychiatric history or symptoms.

In contrast, the variable referred to in the previous manuscript as “mental comorbidity” represented concurrent psychiatric symptom/comorbidity information recorded during the study assessment among participants who were able to complete the protocol. The available dataset primarily contains anxiety- and depressive-symptom categories within this variable.

We have revised the Methods to distinguish clearly between:

(1) the exclusion criterion,

(2) previous psychiatric history, and

(3) concurrent non-PAD psychiatric symptoms/comorbidity recorded during the assessment.

We have also avoided implying that every concurrent symptom category represented an independently confirmed ICD-10 psychiatric diagnosis.

**Comment 9: Which QoL instrument/measure did you use?**

**Response 9:** We thank you for identifying this important ambiguity.

A validated dermatology-specific or acne-specific quality-of-life questionnaire was not used for this variable.

The study case-report form recorded acne-related life impact using three predefined categories: little, moderate, or major impact. For the primary regression model, major impact was contrasted with little/moderate impact.

We have therefore replaced wording suggesting the use of a validated QoL instrument with the more accurate term “study-specific acne-related life impact.”

The absence of a validated acne-specific quality-of-life instrument is now explicitly acknowledged as a limitation.

**Comment 10: Is the Ethics approval number 130 or 131? Both are mentioned different places.**

**Response 10:** Thank you for identifying this inconsistency.

We carefully rechecked and standardized the ethics information throughout the revised manuscript.

The correct approval information is:

**No. 131/HĐĐĐ-BVDLTW, dated 27 November 2025.**

All inconsistent references to another approval number have been corrected.

**Comment 11: The next to last 2 paragraphs of the Introduction have no references to support the statements given there. Most of the statements in the discussion are not supported by references.**

**Response 11:** We agree and have extensively revised both sections.

Additional references have been added to support statements concerning the psychological burden of acne, social-evaluative concerns, stigma, anxiety, quality-of-life impairment, psychodermatology, and relevant clinical implications.

We have also revised or removed broad culture-specific statements that could not be directly supported by the present study or appropriate literature. The Vietnamese setting is now described primarily as an understudied clinical context rather than as evidence of specific cultural causal mechanisms.

Similarly, mechanistic interpretations in the Discussion concerning social scrutiny, avoidance, scarring, treatment experience, and psychological vulnerability are now either supported by references or explicitly presented as hypotheses rather than established causal mechanisms.

**Comment 12: The Title in Table 2 is somewhat generic. Please add ‘..in patients with acne..’ The same applies also to Titles of other tables.**

**Response 12:** We agree and have revised the titles of all main tables to make the study population explicit.

The revised titles are:

**Table 1.** Participant characteristics according to phobic anxiety disorder status among patients with acne vulgaris.

**Table 2.** Prevalence and clinical subtypes of phobic anxiety disorders among patients with acne vulgaris.

**Table 3.** Fear Questionnaire scores among patients with acne vulgaris and phobic anxiety disorders.

**Table 4.** Univariable associations with phobic anxiety disorders among patients with acne vulgaris.

**Table 5.** Multivariable associations with phobic anxiety disorders among patients with acne vulgaris.

**Comment 13: There is a lot of repetition. The conclusion states facts that are described several times previously both in the results and discussion.**

**Response 13:** We agree.

We have shortened the Conclusion and removed repeated numerical findings already presented in the Results and Discussion.

The revised Conclusion now focuses on the central clinical message, the prominence of social phobia, and the need for cautious interpretation and external validation rather than repeating the individual adjusted odds ratios.

**--------------**

**Dear Reviewer #2,**

Thank you for your thoughtful and constructive comments. We have carefully reviewed and revised our manuscript accordingly. We have also highlighted the changes in Bright Green for your convenience. Here are the details:

**Comment: There are some limitations:**

- **The fact that more than 50% are severe cases, expecting higher deterioration of quality of life**
- **More than 60% are in isotretinoin therapy, also revealing the severity, the impact on quality of life.**
- **Almost 60% of patients had poor treatment response.**

**All these limitations could be related to higher phobic traits.**

**Response:** We fully agree with this interpretation and have substantially expanded the corresponding Discussion and limitations.

In our sample, 51.1% of participants were classified as having severe acne, 62.1% were currently receiving isotretinoin, and 57.5% had poor treatment response. These characteristics likely reflect the tertiary referral setting and suggest enrichment for patients with persistent, treatment-experienced, or clinically complex acne.

Such enrichment may be associated with greater psychosocial burden and could contribute to the relatively high proportion of PADs observed in this hospital-based sample.

We now explicitly state that the observed PAD proportion should not be generalized to all patients with acne or interpreted as a population prevalence estimate.

We also acknowledge that severe acne, isotretinoin treatment, treatment response, scarring, and psychological burden may be interrelated rather than independent phenomena.

In the adjusted model, severe acne and current isotretinoin use were not independently associated with PADs, whereas poor treatment response remained associated. Nevertheless, because the study was cross-sectional, this association cannot establish causal direction. Poor treatment response could contribute to psychological distress, but PAD-related avoidance, adherence, healthcare utilization, or perception of treatment benefit could also influence reported response.

We have revised the manuscript to emphasize these alternative explanations and the potential for referral-selection, spectrum bias, residual confounding, and reverse causation.

**-------------**

**Dear Reviewer #3,**

Thank you for your thoughtful and constructive comments. We have carefully reviewed and revised our manuscript accordingly. We have also highlighted the changes in Turquoise for your convenience. Here are the details:

**Comment 1: The Introduction is well structured and provides an appropriate rationale for the study. However, several statements, particularly those concerning the psychological and social consequences of acne and the influence of cultural factors, would benefit from additional supporting references.**

**Response 1:** We agree.

Additional references have been added to support statements concerning psychological morbidity, social anxiety, stigma, social-evaluative concerns, and acne-related impairment.

We have also revised culture-related statements more cautiously. Where direct evidence specific to the Vietnamese context was unavailable, broad assertions concerning cultural norms, family expectations, or help-seeking behavior were removed or reframed.

The revised Introduction now presents Vietnam primarily as an understudied clinical context for clinician-diagnosed PADs rather than attributing the findings to unmeasured cultural mechanisms.

**Comment 2: Although the authors clearly state that PAD diagnoses were established by a qualified psychiatrist according to ICD-10 clinical criteria rather than questionnaire scores alone, additional details regarding the diagnostic procedure would improve reproducibility. Specifically, please clarify whether a structured or semi-structured interview protocol was used, whether all assessments were conducted by the same psychiatrist, and whether any procedures were implemented to ensure diagnostic consistency across participants.**

**Response 2:** We agree and have expanded the description of PAD ascertainment.

PAD diagnoses were established through a psychiatrist-led face-to-face clinical assessment using a study-specific structured case-report form informed by the relevant ICD-10 clinical criteria.

The assessment systematically considered the principal feared situation or trigger, anticipatory anxiety, associated somatic symptoms, avoidance, insight, functional interference, psychiatric history, comorbid symptoms, duration, and alternative explanations.

The Fear Questionnaire was used for additional characterization of phobic symptoms and did not independently determine PAD diagnosis.

The assessment was not based on a validated structured diagnostic research interview such as the SCID or MINI, and we have now made this distinction explicit.

The archived analytic dataset does not contain assessor identifiers that would allow us to retrospectively verify that all 280 assessments were performed by the same individual psychiatrist. We therefore do not make that claim in the revised manuscript.

Similarly, a formal inter-rater reliability study was not performed or retained in the available study documentation. This is now explicitly acknowledged as a limitation concerning diagnostic reproducibility.

**Comment 3: The description of the measurement procedures would benefit from further clarification. While the assessment of phobic anxiety disorders and the Fear Questionnaire are adequately described, it remains unclear how several other psychological and psychosocial variables (e.g., anxious temperament, body-image concern, self-esteem, perceived stigma, and avoidant coping) were assessed.**

**Response 3:** We agree and have substantially revised this section.

The available dataset shows that the psychosocial characteristics used in the study were primarily study-specific categorical items recorded through the structured case-report form and face-to-face clinical assessment rather than independent validated psychometric questionnaires.

For example, the variable previously termed “anxious temperament” was derived from a categorical item assessing emotional stability/anxiety proneness. Avoidant coping was also recorded as a categorical coping tendency.

The source dataset does not contain independent validated measures of self-esteem, body-image concern, social inhibition, or perceived stigma. These terms have therefore been removed where they previously implied independent measured constructs.

Appearance-related stigma or social isolation was recorded only as one possible category within the history of psychological trauma and is no longer presented as a separate validated stigma measure.

We have additionally stated that these study-specific categorical items were not subjected to formal psychometric validation or inter-rater reliability testing. This limitation is particularly important for the anxiety-proneness variable because of its strong adjusted association with PADs and the possibility of construct overlap.

**Comment 4: The Discussion section is relatively brief and could be expanded to provide a more comprehensive interpretation of the findings. In particular, the clinical implications deserve further attention. It would be valuable to discuss how dermatologists might recognize patients who would benefit from further psychiatric or psychological assessment, and whether the use of routine screening tools in dermatology practice could facilitate earlier identification and referral.**

**Response 4:** We agree and have expanded the Discussion accordingly.

The revised manuscript now discusses practical clinical indicators that may prompt additional mental-health assessment in dermatology practice, including marked fear of scrutiny, avoidance of social situations, interference with school/work/relationships, persistent appearance-related concern, scarring, and substantial treatment burden.

We emphasize that brief case-finding questions may help dermatologists recognize patients who require more comprehensive psychological or psychiatric evaluation.

We have also added discussion of the potential role of validated brief social-anxiety screening instruments in dermatology settings. However, we explicitly distinguish screening from diagnosis. A positive screening result should support further clinical assessment or referral rather than establish a PAD diagnosis.

Because the present study did not validate a screening algorithm or diagnostic cut-off for Vietnamese acne patients, we do not recommend a specific screening threshold.

We now propose that future studies should evaluate culturally and linguistically validated screening approaches and integrated dermatology–mental-health referral pathways prospectively.

--- THE END ---
