# Supplementary material for: Phobic anxiety disorders among patients with acne vulgaris at a national dermatology hospital in Vietnam: Prevalence, clinical phenotype, and associated factors
Source: PLoS One. 2026 Sep 10;21(9):e0357803. doi: 10.1371/journal.pone.0357803 (PMC13561362; doi:10.1371/journal.pone.0357803)
Supplement: Peer review history

# Peer review: Decision Letter 0

**DOI:** 10.1371/journal.pone.0357803.r001

**Authors:** Pavel Chernyshov

29 Jul 2026

Dear Dr. Duong,

Thank you for submitting your manuscript to PLOS ONE. After careful consideration, we feel that it has merit but does not fully meet PLOS ONE’s publication criteria as it currently stands. Therefore, we invite you to submit a revised version of the manuscript that addresses the points raised during the review process.

Please submit your revised manuscript by Sep 27 2026 11:59PM. If you will need more time than this to complete your revisions, please reply to this message or contact the journal office at plosone@plos.org. When you're ready to submit your revision, log on to https://www.editorialmanager.com/pone/ and select the 'Submissions Needing Revision' folder to locate your manuscript file.

- A letter that responds to each point raised by the academic editor and reviewer(s). You should upload this letter as a separate file labeled 'Response to Reviewers'.
- A marked-up copy of your manuscript that highlights changes made to the original version. You should upload this as a separate file labeled 'Revised Manuscript with Track Changes'.
- An unmarked version of your revised paper without tracked changes. You should upload this as a separate file labeled 'Manuscript'.

If you would like to make changes to your financial disclosure, please include your updated statement in your cover letter. Guidelines for resubmitting your figure files are available below the reviewer comments at the end of this letter.

If applicable, we recommend that you deposit your laboratory protocols in protocols.io to enhance the reproducibility of your results. Protocols.io assigns your protocol its own identifier (DOI) so that it can be cited independently in the future. For instructions see: https://journals.plos.org/plosone/s/submission-guidelines#loc-laboratory-protocols. Additionally, PLOS ONE offers an option for publishing peer-reviewed Lab Protocol articles, which describe protocols hosted on protocols.io. Read more information on sharing protocols at https://plos.org/protocols?utm_medium=editorial-email&utm_source=authorletters&utm_campaign=protocols.

As the corresponding author, your ORCID iD is verified in the submission system and will appear in the published article. PLOS supports the use of ORCID, and we encourage all coauthors to register for an ORCID iD and use it as well. Please encourage your coauthors to verify their ORCID iD within the submission system before final acceptance, as unverified ORCID iDs will not appear in the published article. *Only*  the individual author can complete the verification step; PLOS staff *cannot*  verify ORCID iDs on behalf of authors.

We look forward to receiving your revised manuscript.

Kind regards,

Pavel V. Chernyshov, M.D., Ph.D.

Academic Editor

PLOS One

Journal Requirements:

When submitting your revision, we need you to address these additional requirements.

1. Please ensure that your manuscript meets PLOS ONE's style requirements, including those for file naming. The PLOS ONE style templates can be found at

https://journals.plos.org/plosone/s/file?id=wjVg/PLOSOne_formatting_sample_main_body.pdf and

https://journals.plos.org/plosone/s/file?id=ba62/PLOSOne_formatting_sample_title_authors_affiliations.pdf

2. We noticed you have some minor occurrence of overlapping text with the following previous publication(s), which needs to be addressed:

https://opus.lib.uts.edu.au/bitstream/10453/168912/2/02whole.pdf

In your revision ensure you cite all your sources (including your own works), and quote or rephrase any duplicated text outside the methods section. Further consideration is dependent on these concerns being addressed.

3. Please provide a complete Data Availability Statement in the submission form, ensuring you include all necessary access information or a reason for why you are unable to make your data freely accessible. If your research concerns only data provided within your submission, please write "All data are in the manuscript and/or supporting information files" as your Data Availability Statement.

4. Please include captions for your Supporting Information files at the end of your manuscript, and update any in-text citations to match accordingly. Please see our Supporting Information guidelines for more information: http://journals.plos.org/plosone/s/supporting-information.

5. If the reviewer comments include a recommendation to cite specific previously published works, please review and evaluate these publications to determine whether they are relevant and should be cited. There is no requirement to cite these works unless the editor has indicated otherwise.

[Note: HTML markup is below. Please do not edit.]

Reviewers' comments:

Reviewer's Responses to Questions

**Comments to the Author**

1. Is the manuscript technically sound, and do the data support the conclusions?

Reviewer #1: Yes

Reviewer #2: Yes

Reviewer #3: Yes

**********

2. Has the statistical analysis been performed appropriately and rigorously?

Reviewer #1: Yes

Reviewer #2: Yes

Reviewer #3: Yes

**********

3. Have the authors made all data underlying the findings in their manuscript fully available?

The PLOS Data policy

Reviewer #1: Yes

Reviewer #2: Yes

Reviewer #3: Yes

**********

4. Is the manuscript presented in an intelligible fashion and written in standard English?

Reviewer #1: Yes

Reviewer #2: Yes

Reviewer #3: Yes

**********

Reviewer #1: The manuscript addresses an interesting and relatively novel question—clinician-diagnosed phobic anxiety disorders (PADs) in patients with acne—which fits well within psychodermatology. The paper is generally well organized, clearly written

The psychosocial variables are poorly defined (anxious temperament, anxious temperament, emotional instability, social inhibition, body-image concern, avoidant coping, self-esteem, perceived stigma). How were these variables assessed? Were validated instruments used, if yes, which and please describe for the dermatological readeship. Or were they rated as clinical impressions? Please provide evidence of validity and reliability. Especially for the variables which gave strong association (anxious temoperament, etc)

'Poor treatment response' is another strong predictor. But what exactly is treatment response? Who evaluated it? Dermatologist? Patient? Both? Objective improvement? IGA? Patient satisfaction? This variable needs a much clearer operational definition.

Please comment on Selection bias. The study is based on one tertiary referral hospital. What about generalizability?

9 variables were evaluated for 69 events. It seems there might be too many variables. CIs are quite unstable. Please mention this in more detail as a limitation.

STROBE recommends a flow diagram of participants. How many were approached? How many refused? How many were excluded and for what reason?

Severity is said to use an IGA-oriented approach instead of a validated grading system. IGA is not given in full. Was this the Investigator's Global Assessment?

Why was ICD-10 chosen instead og DSM?

What is severe psychiatric disorder? And you include mental comorbidity as a covariate. Please elaborate.

Which QoL instrument/measure did you use?

Is the Ethics approval number 130 or 131? Both are mentioned different places

The next to last 2 paragraphs of the Introduction have no references to support the statements given there. Most of the statements in the discussion are not supported by references.

The Title in Table 2 is somewhat generic. Please add '..in patients with acne..' The same applies also to Titles of other tables

There is a lot of repetition. The conclusion states facts that are described several times previously both in the results and discussion.

Reviewer #2: Acne is a common disease in dermatological practice, affecting teenagers and young people mostly. The higher pressure of social media and social environments regarding a perfect image has a huge impact on patients’ quality of life with lots of social phobia aspects. All these issues have to be considered in the approach of acne for better compliance and better results.

There are some limitations:

- The fact that more than 50% are severe cases, expecting higher deterioration of quality of life

- More than 60% are in isotretinoin therapy, also revealing the severity, the impact on quality of life.

- Almost 60% of patients had poor treatment response.

All these limitations could be related to higher phobic traits.

Reviewer #3: 1. The Introduction is well structured and provides an appropriate rationale for the study. However, several statements, particularly those concerning the psychological and social consequences of acne and the influence of cultural factors, would benefit from additional supporting references. Providing citations for these statements would further strengthen the scientific background of the manuscript.

2. Although the authors clearly state that PAD diagnoses were established by a qualified psychiatrist according to ICD-10 clinical criteria rather than questionnaire scores alone, additional details regarding the diagnostic procedure would improve reproducibility. Specifically, please clarify whether a structured or semi-structured interview protocol was used, whether all assessments were conducted by the same psychiatrist, and whether any procedures were implemented to ensure diagnostic consistency across participants.

3. The description of the measurement procedures would benefit from further clarification. While the assessment of phobic anxiety disorders and the Fear Questionnaire are adequately described, it remains unclear how several other psychological and psychosocial variables (e.g., anxious temperament, body-image concern, self-esteem, perceived stigma, and avoidant coping) were assessed. Please specify the measurement method for these variables (e.g., validated questionnaire, structured interview, clinician rating, or study-specific assessment) and provide references where appropriate.

4. The Discussion section is relatively brief and could be expanded to provide a more comprehensive interpretation of the findings. In particular, the clinical implications deserve further attention. It would be valuable to discuss how dermatologists might recognize patients who would benefit from further psychiatric or psychological assessment, and whether the use of routine screening tools in dermatology practice could facilitate earlier identification and referral.

**********

what does this mean?). If published, this will include your full peer review and any attached files.

If you choose “no”, your identity will remain anonymous but your review may still be made public.

**Do you want your identity to be public for this peer review?**  For information about this choice, including consent withdrawal, please see our Privacy Policy

Reviewer #1: **Yes:** Flora Balieva

Reviewer #2: **Yes:** Liana Manolache

Reviewer #3: **Yes:** Jutta Major

**********

[NOTE: If reviewer comments were submitted as an attachment file, they will be attached to this email and accessible via the submission site. Please log into your account, locate the manuscript record, and check for the action link "View Attachments". If this link does not appear, there are no attachment files.]

To ensure your figures meet our technical requirements, please review our figure guidelines: https://journals.plos.org/plosone/s/figures

You may also use PLOS’s free figure tool, NAAS, to help you prepare publication quality figures: https://journals.plos.org/plosone/s/figures#loc-tools-for-figure-preparation.

NAAS will assess whether your figures meet our technical requirements by comparing each figure against our figure specifications.

# Author comment: Author response to Decision Letter 1

**DOI:** 10.1371/journal.pone.0357803.r002

13 Aug 2026

Dear Reviewer #1,

Thank you for your thoughtful and constructive comments. We have carefully reviewed and revised our manuscript accordingly. We have also highlighted the changes in yellow for your convenience. Here are the details:

Comment 1: The psychosocial variables are poorly defined (anxious temperament, anxious temperament, emotional instability, social inhibition, body-image concern, avoidant coping, self-esteem, perceived stigma). How were these variables assessed? Were validated instruments used, if yes, which and please describe for the dermatological readership. Or were they rated as clinical impressions? Please provide evidence of validity and reliability. Especially for the variables which gave strong association (anxious temperament, etc).

Response 1: We agree with you that the previous description was insufficient and could incorrectly imply that all psychosocial constructs were measured using validated psychometric instruments.

We have therefore substantially revised the Methods and terminology. The available study data show that these characteristics were recorded as study-specific categorical items in the structured case-report form and face-to-face clinical assessment rather than as separate validated psychometric scales.

In particular, the variable previously termed “anxious temperament” was derived from a case-report-form item concerning emotional stability/anxiety proneness. Participants categorized as “easily anxious” were contrasted with those categorized as stable/carefree or relatively stable. Avoidant coping was similarly recorded as a categorical coping tendency.

The source dataset does not contain independent validated scales for self-esteem, body-image concern, social inhibition, or perceived stigma. We have therefore removed wording suggesting that these were independently measured psychosocial constructs.

Importantly, no study-specific psychometric validation or inter-rater reliability evaluation was performed for these categorical clinical items. We now state this explicitly as a limitation. We have also revised the Discussion to emphasize that the strong association observed for anxiety proneness should be interpreted cautiously because of potential construct overlap with PAD symptoms and possible common-method bias.

Comment 2: ‘Poor treatment response’ is another strong predictor. But what exactly is treatment response? Who evaluated it? Dermatologist? Patient? Both? Objective improvement? IGA? Patient satisfaction? This variable needs a much clearer operational definition.

Response 2: We agree and have clarified this variable substantially.

Treatment response was recorded in the structured study case-report form using three categories: no response, partial response, and marked response. For the primary regression analysis, no response and partial response were combined as “poor treatment response” and compared with marked response.

This variable was recorded as part of the clinical assessment, treatment history, and medical-record review. The available analytic data do not identify a separate assessor-specific field that would allow us to classify the measure retrospectively as exclusively dermatologist-rated or patient-rated.

Importantly, treatment response was not defined using a standardized longitudinal Investigator’s Global Assessment change, percentage reduction in lesion count, or a validated patient-satisfaction instrument. We have revised the Methods accordingly and now describe it explicitly as a study-specific clinical treatment-response classification.

We have also strengthened the Discussion and limitations to acknowledge possible measurement error, subjective components of treatment-response assessment, and the inability of the cross-sectional design to establish whether poor response contributed to PADs or whether psychological factors influenced treatment experience, adherence, or reporting.

Comment 3: Please comment on Selection bias. The study is based on one tertiary referral hospital. What about generalizability?

Response 3: We agree and have considerably expanded the discussion of selection and spectrum bias.

The study used convenience sampling at a single national tertiary dermatology hospital. The sample consequently contained a substantial proportion of patients with clinically complex acne: 51.1% had severe acne, 62.1% were receiving isotretinoin, and 57.5% had poor treatment response.

These characteristics may reflect referral of patients with more severe, persistent, or treatment-experienced disease and could contribute to a higher psychological burden than would be expected in community or primary-care acne populations.

We therefore now explicitly state that the observed 24.6% proportion of PADs is a hospital-based sample proportion and should not be interpreted as a national or population prevalence estimate.

We have also added that adjustment for measured clinical characteristics cannot eliminate referral-selection or spectrum bias. Multicentre studies incorporating primary dermatology, general clinical, and community settings are needed to assess external validity.

Comment 4: 9 variables were evaluated for 69 events. It seems there might be too many variables. CIs are quite unstable. Please mention this in more detail as a limitation.

Response 4: We agree.

The primary multivariable model contained nine covariates and 69 PAD events, corresponding to approximately 7.7 events per modeled covariate. We now report this explicitly and describe the multivariable analysis as exploratory.

We have expanded the limitations to acknowledge the risk of overfitting, coefficient instability, and reduced precision associated with the modest event count. Interpretation now emphasizes effect estimates and 95% confidence intervals rather than statistical significance alone.

The sensitivity analyses are retained to evaluate the robustness of the main findings, but we explicitly state that they do not eliminate the potential for model instability. In particular, the very large estimate observed when treatment response was modeled in three levels is now clearly described as evidence of sparse data/near-separation and not as a precise effect estimate.

Comment 5: STROBE recommends a flow diagram of participants. How many were approached? How many refused? How many were excluded and for what reason?

Response 5: We agree that complete recruitment-flow information would strengthen reporting.

The available analytic dataset contains the 280 participants who completed the PAD assessment. However, a separate screening log documenting the total number approached, refusing participation, or excluded at each stage was not retained in the archived study files available for the present analysis.

We therefore cannot retrospectively reconstruct these numbers accurately without introducing unverifiable information. For this reason, we have not generated a numerical flow diagram containing estimated or inferred recruitment counts.

Instead, we have clarified the convenience-sampling procedure in the Methods and added incomplete reporting of recruitment flow and response rate as a limitation.

Comment 6: Severity is said to use an IGA-oriented approach instead of a validated grading system. IGA is not given in full. Was this the Investigator's Global Assessment?

Response 6: Yes. We thank you for identifying this ambiguity.

We have now defined IGA in full as Investigator’s Global Assessment.

We have also clarified that the analytic dataset contains a three-category clinician global acne severity classification—mild, moderate, or severe—rather than a formal numerical validated IGA score.

The Methods have therefore been revised to avoid implying that a fully standardized numerical IGA instrument was prospectively recorded.

Comment 7: Why was ICD-10 chosen instead of DSM?

Response 7: We have now added this clarification to the Methods.

ICD-10 was the prespecified diagnostic framework used in the study protocol for psychiatric assessment. PAD diagnoses were based on psychiatrist-led clinical assessment against ICD-10 clinical criteria rather than questionnaire thresholds alone.

Because the study protocol was designed around ICD-10 diagnostic categories, DSM criteria were not applied retrospectively as a parallel diagnostic system. We have clarified this rationale in the revised manuscript.

Comment 8: What is severe psychiatric disorder? And you include mental comorbidity as a covariate. Please elaborate.

Response 8: We agree that the previous wording did not adequately distinguish these concepts.

The exclusion criterion referred to a previously diagnosed psychiatric disorder of sufficient current severity to substantially interfere with valid participation in the clinical interview and study assessment. It was not intended to exclude all participants with any psychiatric history or symptoms.

In contrast, the variable referred to in the previous manuscript as “mental comorbidity” represented concurrent psychiatric symptom/comorbidity information recorded during the study assessment among participants who were able to complete the protocol. The available dataset primarily contains anxiety- and depressive-symptom categories within this variable.

We have revised the Methods to distinguish clearly between:

(1) the exclusion criterion,

(2) previous psychiatric history, and

(3) concurrent non-PAD psychiatric symptoms/comorbidity recorded during the assessment.

We have also avoided implying that every concurrent symptom category represented an independently confirmed ICD-10 psychiatric diagnosis.

Comment 9: Which QoL instrument/measure did you use?

Response 9: We thank you for identifying this important ambiguity.

A validated dermatology-specific or acne-specific quality-of-life questionnaire was not used for this variable.

The study case-report form recorded acne-related life impact using three predefined categories: little, moderate, or major impact. For the primary regression model, major impact was contrasted with little/moderate impact.

We have therefore replaced wording suggesting the use of a validated QoL instrument with the more accurate term “study-specific acne-related life impact.”

The absence of a validated acne-specific quality-of-life instrument is now explicitly acknowledged as a limitation.

Comment 10: Is the Ethics approval number 130 or 131? Both are mentioned different places.

Response 10: Thank you for identifying this inconsistency.

We carefully rechecked and standardized the ethics information throughout the revised manuscript.

The correct approval information is:

No. 131/HĐĐĐ-BVDLTW, dated 27 November 2025.

All inconsistent references to another approval number have been corrected.

Comment 11: The next to last 2 paragraphs of the Introduction have no references to support the statements given there. Most of the statements in the discussion are not supported by references.

Response 11: We agree and have extensively revised both sections.

Additional references have been added to support statements concerning the psychological burden of acne, social-evaluative concerns, stigma, anxiety, quality-of-life impairment, psychodermatology, and relevant clinical implications.

We have also revised or removed broad culture-specific statements that could not be directly supported by the present study or appropriate literature. The Vietnamese setting is now described primarily as an understudied clinical context rather than as evidence of specific cultural causal mechanisms.

Similarly, mechanistic interpretations in the Discussion concerning social scrutiny, avoidance, scarring, treatment experience, and psychological vulnerability are now either supported by references or explicitly presented as hypotheses rather than established causal mechanisms.

Comment 12: The Title in Table 2 is somewhat generic. Please add ‘..in patients with acne..’ The same applies also to Titles of other tables.

Response 12: We agree and have revised the titles of all main tables to make the study population explicit.

The revised titles are:

Table 1. Participant characteristics according to phobic anxiety disorder status among patients with acne vulgaris.

Table 2. Prevalence and clinical subtypes of phobic anxiety disorders among patients with acne vulgaris.

Table 3. Fear Questionnaire scores among patients with acne vulgaris and phobic anxiety disorders.

Table 4. Univariable associations with phobic anxiety disorders among patients with acne vulgaris.

Table 5. Multivariable associations with phobic anxiety disorders among patients with acne vulgaris.

Comment 13: There is a lot of repetition. The conclusion states facts that are described several times previously both in the results and discussion.

Response 13: We agree.

We have shortened the Conclusion and removed repeated numerical findings already presented in the Results and Discussion.

The revised Conclusion now focuses on the central clinical message, the prominence of social phobia, and the need for cautious interpretation and external validation rather than repeating the individual adjusted odds ratios.

--------------

Dear Reviewer #2,

Thank you for your thoughtful and constructive comments. We have carefully reviewed and revised our manuscript accordingly. We have also highlighted the changes in Bright Green for your convenience. Here are the details:

Comment: There are some limitations:

• The fact that more than 50% are severe cases, expecting higher deterioration of quality of life

• More than 60% are in isotretinoin therapy, also revealing the severity, the impact on quality of life.

• Almost 60% of patients had poor treatment response.

All these limitations could be related to higher phobic traits.

Response: We fully agree with this interpretation and have substantially expanded the corresponding Discussion and limitations.

In our sample, 51.1% of participants were classified as having severe acne, 62.1% were currently receiving isotretinoin, and 57.5% had poor treatment response. These characteristics likely reflect the tertiary referral setting and suggest enrichment for patients with persistent, treatment-experienced, or clinically complex acne.

Such enrichment may be associated with greater psychosocial burden and could contribute to the relatively high proportion of PADs observed in this hospital-based sample.

We now explicitly state that the observed PAD proportion should not be generalized to all patients with acne or interpreted as a population prevalence estimate.

We also acknowledge that severe acne, isotretinoin treatment, treatment response, scarring, and psychological burden may be interrelated rather than independent phenomena.

In the adjusted model, severe acne and current isotretinoin use were not independently associated with PADs, whereas poor treatment response remained associated. Nevertheless, because the study was cross-sectional, this association cannot establish causal direction. Poor treatment response could contribute to psychological distress, but PAD-related avoidance, adherence, healthcare utilization, or perception of treatment benefit could also influence reported response.

We have revised the manuscript to emphasize these alternative explanations and the potential for referral-selection, spectrum bias, residual confounding, and reverse causation.

-------------

Dear Reviewer #3,

Thank you for your thoughtful and constructive comments. We have carefully reviewed and revised our manuscript accordingly. We have also highlighted the changes in Turquoise for your convenience. Here are the details:

Comment 1: The Introduction is well structured and provides an appropriate rationale for the study. However, several statements, particularly those concerning the psychological and social consequences of acne and the influence of cultural factors, would benefit from additional supporting references.

Response 1: We agree.

Additional references have been added to support statements concerning psychological morbidity, social anxiety, stigma, social-evaluative concerns, and acne-related impairment.

We have also revised culture-related statements more cautiously. Where direct evidence specific to the Vietnamese context was unavailable, broad assertions concerning cultural norms, family expectations,

Attachment Submitted filename: Response to reviewers.docx

# Peer review: Decision Letter 1

**DOI:** 10.1371/journal.pone.0357803.r003

**Authors:** Pavel Chernyshov

23 Aug 2026

Phobic anxiety disorders among patients with acne vulgaris at a national dermatology hospital in Vietnam: prevalence, clinical phenotype, and associated factors

PONE-D-26-35566R1

Dear Dr. Mai Son Duong,

We’re pleased to inform you that your manuscript has been judged scientifically suitable for publication and will be formally accepted for publication once it meets all outstanding technical requirements.

Within one week, you’ll receive an e-mail detailing the required amendments. When these have been addressed, you’ll receive a formal acceptance letter and your manuscript will be scheduled for publication.

An invoice will be generated when your article is formally accepted. Please note, if your institution has a publishing partnership with PLOS and your article meets the relevant criteria, all or part of your publication costs will be covered. Please make sure your user information is up-to-date by logging into Editorial Manager at Editorial Manager® and clicking the ‘Update My Information' link at the top of the page. For questions related to billing, please contact billing support.

If your institution or institutions have a press office, please notify them about your upcoming paper to help maximize its impact. If they’ll be preparing press materials, please inform our press team as soon as possible -- no later than 48 hours after receiving the formal acceptance. Your manuscript will remain under strict press embargo until 2 pm Eastern Time on the date of publication. For more information, please contact onepress@plos.org.

Kind regards,

Pavel V. Chernyshov, M.D., Ph.D.

Academic Editor

PLOS One

Additional Editor Comments (optional):

Reviewers' comments:

Reviewer's Responses to Questions

**Comments to the Author**

Reviewer #1: All comments have been addressed

Reviewer #2: (No Response)

**********

2. Is the manuscript technically sound, and do the data support the conclusions?

Reviewer #1: Yes

Reviewer #2: No

**********

3. Has the statistical analysis been performed appropriately and rigorously?

Reviewer #1: Yes

Reviewer #2: Yes

**********

4. Have the authors made all data underlying the findings in their manuscript fully available?

The PLOS Data policy

Reviewer #1: Yes

Reviewer #2: Yes

**********

5. Is the manuscript presented in an intelligible fashion and written in standard English?

Reviewer #1: Yes

Reviewer #2: Yes

**********

Reviewer #1: Although it seems that the authors have addressed all comments adequately a true tracked version is not available, making assessment of true changes difficult.

Whole paragraphs are highlighted, and the whole Discussion and Conclusions are highlighted with no indication what the changes made are. It is not visible what was removed and what was added, how the text has been edited.

Since the document now is 72 pages long, a detailed readthrough to assess all changes was not feasible for this reviewer.

For a true assessment of changes, I suggest the authors use a tracking function.

Besides, some comments overlap between reviewers, yet changes are strictly separated by reviewer.

This reviewer does not see the value of separating changed text by reviewer since the revisions are assessed as a whole, not each reviewer assessing their own comments only.

No changes in references are marked. Were all identical to the first version although text was removed and other added?

Reviewer #2: There were many comments and observations that authors had to manage for the revised paper.

With all the limitations regarding tertiary approach of potential complicated, treatment-resistant cases that come with more emotional difficulties, the paper highlights the importance of psychological evaluation for acne patients for a better therapeutical scheme. It is important for all clinicians to take in consideration the psychological and quality of life impact of all chronic skin disease.

**********

what does this mean?). If published, this will include your full peer review and any attached files.

If you choose “no”, your identity will remain anonymous but your review may still be made public.

**Do you want your identity to be public for this peer review?**  For information about this choice, including consent withdrawal, please see our Privacy Policy

Reviewer #1: **Yes:** Flora Balieva

Reviewer #2: **Yes:** Liana Manolache

**********

# Editor report: Acceptance letter

**DOI:** 10.1371/journal.pone.0357803.r004

**Authors:** Pavel Chernyshov

PONE-D-26-35566R1

PLOS One

Dear Dr. Duong,

I'm pleased to inform you that your manuscript has been deemed suitable for publication in PLOS One. Congratulations! Your manuscript is now being handed over to our production team.

At this stage, our production department will prepare your paper for publication. This includes ensuring the following:

* All references, tables, and figures are properly cited

* All relevant supporting information is included in the manuscript submission,

* There are no issues that prevent the paper from being properly typeset

You will receive further instructions from the production team, including instructions on how to review your proof when it is ready. Please keep in mind that we are working through a large volume of accepted articles, so please give us a few days to review your paper and let you know the next and final steps.

Lastly, if your institution or institutions have a press office, please let them know about your upcoming paper now to help maximize its impact. If they'll be preparing press materials, please inform our press team within the next 48 hours. Your manuscript will remain under strict press embargo until 2 pm Eastern Time on the date of publication. For more information, please contact onepress@plos.org.

You will receive an invoice from PLOS for your publication fee after your manuscript has reached the completed accept phase. If you receive an email requesting payment before acceptance or for any other service, this may be a phishing scheme. Learn how to identify phishing emails and protect your accounts at https://explore.plos.org/phishing.

If we can help with anything else, please email us at customercare@plos.org.

Thank you for submitting your work to PLOS One and supporting open access.

Kind regards,

PLOS One Editorial Office Staff

on behalf of

Dr. Pavel V. Chernyshov

Academic Editor

PLOS One
